# High-Resolution Silicon Photomultiplier Time-of-Flight Dedicated Head PET System for Clinical Brain Studies

**DOI:** 10.2967/jnumed.122.264080

**Published:** 2023-01

**Authors:** Kazunari Ishii, Kohei Hanaoka, Shota Watanabe, Daisuke Morimoto-Ishikawa, Takahiro Yamada, Hayato Kaida, Yoshiyuki Yamakawa, Suzuka Minagawa, Shiho Takenouchi, Atsushi Ohtani, Tetsuro Mizuta

**Affiliations:** 1 Department of Radiology, Kindai University Faculty of Medicine, Osakasayama, Japan;; 2 Division of Positron Emission Tomography, Institute of Advanced Clinical Medicine, Kindai University Hospital, Osakasayama, Japan; and; 3 Medical Systems Division, Shimadzu Corporation, Kyoto, Japan

**Keywords:** high resolution, silicon photomultiplier, time of flight, dedicated head PET, brain

## Abstract

We acquired brain ^18^F-FDG and ^18^F-flutemetamol PET images using a time-of-flight system dedicated to the head (dhPET) and a conventional whole-body PET/CT (wbPET) system and evaluated the clinical superiority of dhPET over wbPET. **Methods:** There were 18 subjects for the ^18^F-FDG PET study and 17 subjects for the ^18^F-flutemetamol PET study. ^18^F-FDG PET images were first obtained using wbPET, followed by dhPET. ^18^F-flutemetamol PET images were first obtained using wbPET, followed by dhPET. Images acquired using dhPET and wbPET were compared by visual inspection, voxelwise analysis, and SUV ratio (SUVR). **Results:** All ^18^F-FDG and ^18^F-flutemetamol images acquired using dhPET were judged as visually better than those acquired using wbPET. The voxelwise analysis demonstrated that accumulations in the cerebellum, in the lateral occipital cortices, and around the central sulcus area in dhPET ^18^F-FDG images were lower than those in wbPET ^18^F-FDG images, whereas accumulations around the ventricle systems were higher in dhPET ^18^F-FDG images than those in wbPET ^18^F-FDG images. Accumulations in the cerebellar dentate nucleus, in the midbrain, in the lateral occipital cortices, and around the central sulcus area in dhPET images were lower than those in wbPET images, whereas accumulations around the ventricle systems were higher in dhPET ^18^F-flutemetamol images than those in wbPET ^18^F-flutemetamol images. The mean cortical SUVRs of ^18^F-FDG and ^18^F-flutemetamol dhPET images were significantly higher than those of ^18^F-FDG and ^18^F-flutemetamol wbPET images, respectively. **Conclusion:** The dhPET images had better image quality by visual inspection and higher SUVRs than wbPET images. Although there were several regional accumulation differences between dhPET and wbPET images, understanding this phenomenon will enable full use of the features of this dhPET system in clinical practice.

## Visual Abstract

**Figure:**
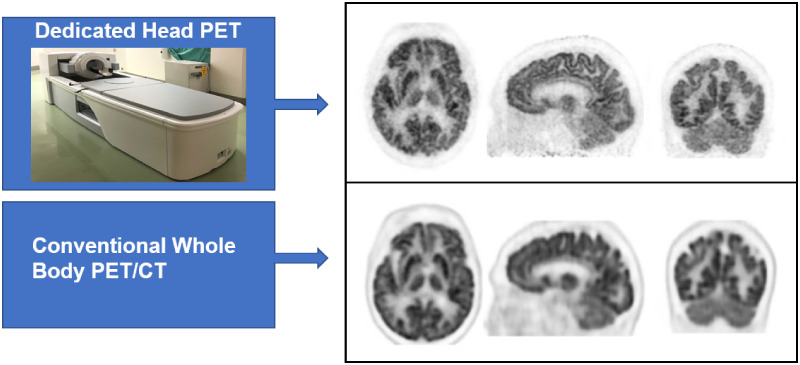

The field of brain PET has recognized the importance of ^18^F-FDG PET, amyloid PET, and tau PET scans for diagnosing dementia. This recognition reflects the increase in the number of dementia patients due to an aging society, with the number of scans expected to increase (1–4). Whole-body PET (wbPET)/CT scanners are not optimal for imaging small structures such as the brain; conventional wbPET scanners are large and expensive, and their spatial resolution is not always sufficient for brain examinations. Ideally, the spatial resolution should be sufficient to enable delineation of the thickness of the gray matter and small brain structures without partial-volume effects. There are several head-only–designed PET scanners, such as HRRT (CTI/Siemens) (5), NEUROLOGICAL PET/CT (Photo Diagnostic Systems) (6), brain PET (Hamamatsu Photonics K.K.) (7), and brain-dedicated helmet-type PET (Vrain; Atox Co., Ltd.) (8). They have been reported to have better spatial resolution than conventional wbPET scanners. Catana reviewed the development of these dedicated head PET (dhPET) imaging devices and expects further improvements to be made: improvements in imaging smaller structures, such as hippocampal subfields and thalamic and brain stem nuclei; improvements in sensitivity without sacrificing spatial resolution; improvements in the portability, mobility, and wearability of the device; and reductions in the cost of the scanner (9). Thus, our collaborators modified a dedicated breast PET scanner—a silicon photomultiplier time-of-flight (TOF) dhPET PET scanner (SET-5002; Shimadzu Corp.)—to enable its use not only for breast imaging but also for brain imaging. Detailed specifications of the dhPET system are described elsewhere (10). This TOF PET system for the head and breast is designed to be less expensive than the conventional wbPET system but with higher sensitivity and spatial resolution. Although previous dhPET systems have been recognized for their high spatial resolution of ^18^F-FDG PET images, to our knowledge there have been no reports of cases in which diagnoses were clinically overturned and no reports of their implementation in amyloid PET studies. We acquired ^18^F-FDG and amyloid PET data using a conventional wbPET system and our novel brain TOF PET system in the same individuals and compared clinical interpretations and PET tracer uptake between the 2 scanners.

## MATERIALS AND METHODS

### Outline of the New dhPET Scanner

**FIGURE 1.**
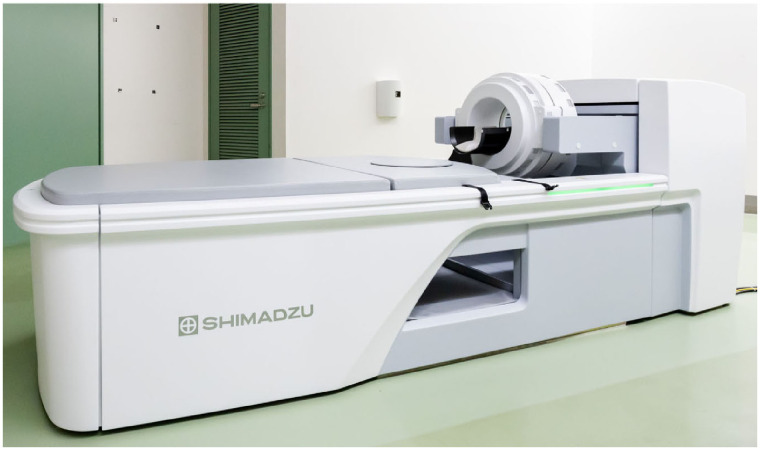
Appearance of dhPET scanner.

Figure 1 shows the appearance of the scanner for brain scan mode. The scanner consisted of 3 detector rings with a diameter of 300 mm, with each ring comprising 16 detector modules, which offer a sufficient axial field of view (FOV), 162 mm, to allow whole-brain scanning. A 3-dimensional image was reconstructed at an isotropic voxel size of 1.1 mm with a matrix of 240 × 240 × 148 using the list-mode dynamic row-action maximum-likelihood algorithm. In brain mode, attenuation correction was performed using the maximum-likelihood attenuation correction factor method combined with the quantification process, which compensates for nonuniformity in the head using the TOF information without CT. First, a nonquantitative μ-map was reconstructed from the attenuation correction factor obtained using the maximum-likelihood attenuation correction factor method. Next, the maximum area of the head in the μ-map was quantified, taking into account the fact that the human head consists primarily of soft tissue, and combined with the structural information of the headrest. Finally, an attenuation-corrected diagnostic image was reconstructed using the μ-map. The dhPET has high spatial resolution and achieves a 2.5-mm full width at half maximum (FWHM) 10 mm from the center of the FOV in NEMA NU 2-2012 (10). In addition, an image of the mini-Derenzo phantom showed that 1.6-mm diameter hot rods could clearly be separated, which visually confirmed the high spatial resolution of dhPET (https://www.shimadzu.com/med/products/pet/brestome.html).

### Subjects

The subjects of this study were those who underwent conventional wbPET imaging for clinical examination, for free medical treatment, or as part of the Japan Agency for Medical Research and Development (AMED) study (jRCTs031180219), who agreed to undergo additional imaging with the dhPET system and allow the use of their wbPET imaging data for this study.

There were 18 subjects for the ^18^F-FDG study (8 men and 10 women): 7 had mild cognitive impairment, 4 had Alzheimer disease, 1 had dementia with Lewy bodies, 1 had frontotemporal dementia, 1 had subjective cognitive impairment, 2 had epilepsy, 1 had lymphoma, and 1 had skull bone metastasis. The mean age of the participants was 67.7 ± 16.0 y.

For the ^18^F-flutemetamol study, we included 17 subjects (8 men and 9 women): 7 with mild cognitive impairment, 4 with Alzheimer disease, 3 healthy older adult subjects, 1 with dementia with Lewy bodies, 1 with frontotemporal dementia, and 1 with subjective cognitive impairment. The mean age of the participants was 73.1 ± 7.6 y. Fourteen subjects participated in both the ^18^F-FDG and the ^18^F-flutemetamol PET studies.

The study protocol was submitted to and approved by the Certified Review Board of Hyogo College of Medicine (jRCTs052200055). Our institutional review board also approved this study, and written informed consent was obtained from all participants.

### Image Acquisition and Reconstruction

For the ^18^F-FDG PET scan, the subjects fasted for 4 h before being administered ^18^F-FDG. The mean dose was 192.0 ± 18.7 MBq (range, 150.5–213.4 MBq). ^18^F-FDG PET imaging of the brain was performed using the Discovery PET/CT 710 scanner (GE Healthcare) for wbPET. The Discovery PET/CT 710 is a combination of a lutetium-based scintillator with a photomultiplier tube PET component and a 16-slice CT component. This scanner enables a 150.42-mm axial FOV and a 700-mm transaxial FOV with 47 image planes spaced at 3.27-mm intervals. The spatial resolution was 5.27 mm at FWHM according to a NEMA NU 2-2007.

The scanning protocol was performed using the Japanese Alzheimer Disease Neuroimaging Initiative ^18^F-FDG PET protocol (11) for cognitive disorders. A 30-min list-mode emission scan was acquired on a wbPET scanner, which started 30 min after the intravenous injection of ^18^F-FDG. Subjects were instructed to lie quietly in a dimly lit room with their eyes open under minimal sensory stimulation. Subsequently, dhPET scanning was performed for 30 min with list mode (average scanning time after injection, 71 ± 5 min). Oncology subjects underwent 2 min of wbPET scanning 60 min after the intravenous injection, followed by 5 min of dhPET scanning (average scanning time after injection, 90 ± 1 min). Epilepsy subjects underwent 20 min of wbPET scanning 60 min after the intravenous injection, followed by 20 min of dhPET scanning (average scanning time after injection, 86 ± 1 min).

The amyloid PET scanning methods with ^18^F-flutemetamol have been described previously (12). The mean dose of ^18^F-flutemetamol was 199.5 ± 9.1 MBq (range, 180.6–210.1 MBq), which was injected intravenously into an antecubital vein. A 20-min list-mode PET scan was acquired from 90 min using the wbPET scanner following the protocol of the AMED study (jRCTsO31180219). Subsequently, a 20-min list-mode scan was obtained using the dhPET system (average scanning time after injection, 117 ± 2 min).

For the wbPET reconstruction conditions, ^18^F-FDG PET images were reconstructed using the following algorithms and conditions: a block sequential regularized expectation-maximization algorithm, β of 100, 256 × 256 matrix, 300-mm transaxial FOV, and 1.2 mm/pixel. ^18^F-flutemetamol PET data were reconstructed using the following algorithms and conditions: 3-dimensional ordered-subsets expectation maximization with TOF, 4 iterations, 16 subsets, 128 × 128 matrix, 256-mm transaxial FOV, 2.0 mm/pixel, and 4.0-mm gaussian filter (FWHM).

For the dhPET reconstruction conditions, ^18^F-FDG PET images were reconstructed using the following algorithms and conditions: list-mode dynamic row action maximum likelihood algorithm, subset of 200, β of 200, 1 iteration, 240 × 240 matrix, 264-mm transaxial FOV, and 1.1 mm/pixel. ^18^F-flutemetamol PET data were reconstructed using the following algorithms and conditions: list-mode dynamic row action maximum-likelihood algorithm, subset of 150, β of 100, 1 iteration, 240 × 240 matrix, 264-mm transaxial FOV, and 1.1 mm/pixel.

These parameters met the criteria for phantom testing based on the PET imaging site qualification program of the Japanese Society of Nuclear Medicine (specifically, the percentage contrast in the Hoffman 3-dimensional brain phantom was greater than 55%, and the coefficient of variation in the cylindric phantom was less than 15%).

### Data Analysis

Spatial resolution was compared visually between the wbPET and dhPET images. The advantage of dhPET over wbPET was scored as follows: 1 (inferior): spatial resolution was lower, or there was low contrast between the lesioned and normal area; 2 (intermediate): spatial resolution was almost equal, and the contrast was equal between the lesioned and normal area; 3 (superior): spatial resolution was higher, or there was high contrast between the lesioned and normal area. Additionally, we examined whether interpretation of the dhPET image changed the clinical diagnosis that had been determined by interpretation of the wbPET image.

First, 2 nuclear medicine physicians independently scored the images to determine interobserver variability. If the 2 physicians disagreed, they discussed their interpretations and determined a final score.

For the voxel-based comparison we used SPM, version 12 (https://www.fil.ion.ucl.ac.uk/spm/software/spm12/). The ^18^F-FDG and ^18^F-flutemetamol images were each coregistered to individual MR images, which were segmented into gray matter, white matter, and cerebrospinal fluid using the SPM12 segmentation program. Individual gray matter images were then spatially normalized to the template image using the “diffeomorphic anatomic registration through exponentiated lie algebra” algorithm (13), and the normalized parameters were applied to the coregistered PET image. PET images were then spatially normalized to Montreal Neurologic Institute space. All images were smoothed using an 8-mm gaussian filter. We then performed a voxel-based comparison using a paired *t* test between the wbPET and dhPET images. The significance threshold was set at a *P* value of less than 0.05 using familywise error correction.

Mean cortical SUV ratios (SUVRs) for each subject for both dhPET and wbPET images were calculated using centiloid volumes of interest: voi_CerebGry_2 mm and voi_ctx_2 mm (http://www.gaain.org/centiloid-project). For regional SUVR, we used 4 volumes of interest in the major regions specific to Alzheimer disease (frontal, temporal, posterior cingulate, and parietal cortices), which were produced for a previous study (14). A paired *t* test was used to compare the mean cortical SUVRs of the ^18^F-FDG and ^18^F-flutemetamol images between dhPET and wbPET acquisitions. The Shapiro–Wilk test revealed that the distribution was not normal in any of the regional SUVRs. Comparisons of regional SUVRs between the 2 scanners for the ^18^F-FDG and ^18^F-flutemetamol images were performed using the nonparametric repeated-measures Friedman test, and post hoc tests were corrected using Bonferroni adjustment.

## RESULTS

**FIGURE 2.**
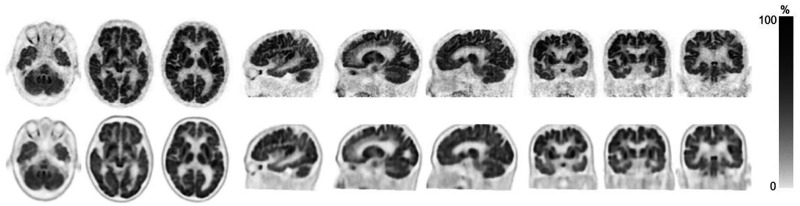
Representative ^18^F-FDG PET image acquired on dhPET scanner (top row) and conventional wbPET scanner (bottom row). Patient is 85-y-old man with mild cognitive impairment due to Alzheimer disease. Bilateral hypometabolism of posterior cingulate gyri, precuneus, and temporal lobe is shown.

**FIGURE 3.**
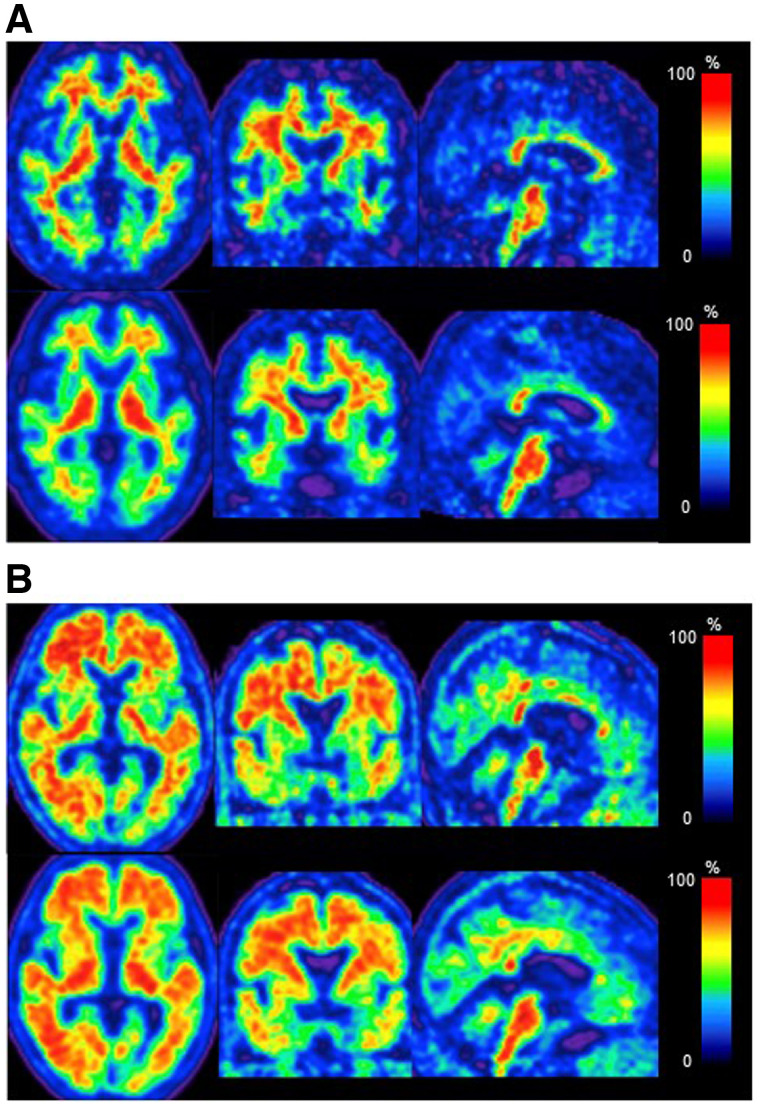
Representative ^18^F-flutemetamol PET images acquired using dhPET scanner (top rows) and conventional wbPET scanner (bottom rows). (A) Amyloid-negative images of 72-y-old healthy man. (B) Amyloid-positive images of 70-y-old male Alzheimer disease patient.

**FIGURE 4.**
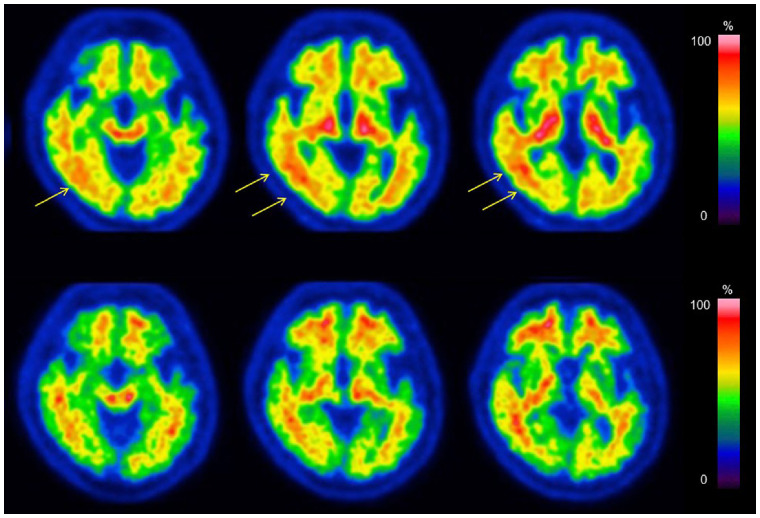
Equivocal case of amyloid accumulation using dhPET scanner and wbPET scanner. (Top row) ^18^F-flutemetamol image acquired using conventional wbPET scanner. (Bottom row) ^18^F-flutemetamol image acquired using dbPET scanner. Patient is 67-y-old man with subjective cognitive impairment. At first glance, patient appeared to be amyloid-negative. However, accumulation in right temporal cortex (arrows) was suspected on wbPET image, whereas dhPET image clearly has no evidence of accumulation in right temporal cortex.

Interobserver agreement on the visual inspection scores was 100% for both ^18^F-FDG and ^18^F-flutemetamol images. The 2 observers scored 3 (superior) for all ^18^F-FDG and ^18^F-flutemetamol images. Figures 2 and 3 show representative ^18^F-FDG and ^18^F-flutemetamol images from both scanners. As shown in Figure 2, the red nucleus was clearly depicted in the dhPET image. For the ^18^F-FDG PET images, the pattern of abnormal uptake in dementia patients was similar across both dhPET and wbPET images. However, the contrast between the decreased and preserved areas was clearer in the dhPET images. In both cases of epilepsy, medial temporal metabolic reduction was observed in both the wbPET and the dhPET images, although the detection of findings was slightly easier on the dhPET images because of the higher resolution. In the malignant tumor cases, the lesions were similarly identifiable, although the dhPET images showed a finer distribution of accumulation than the wbPET images. For the ^18^F-flutemetamol PET images, the detection of amyloid deposition was similar across both PET systems, except for 1 of the 17 subjects, a patient for whom the dhPET image showed a more detailed amyloid distribution. This amyloid PET image of this equivocal case is shown in Figure 4: amyloid accumulation was suspected in the right lateral temporal cortices on the ^18^F-flutemetamol PET image scanned using the wbPET scanner. However, the ^18^F-flutemetamol PET image scanned using the dhPET system showed no cortical accumulation, which indicated an amyloid-negative case.

The mean SUVRs of the dhPET ^18^F-FDG and ^18^F-flutemetamol images were significantly higher than those of the wbPET ^18^F-FDG and ^18^F-flutemetamol images (Table 1). The regional SUVRs of the dhPET ^18^F-FDG and ^18^F-flutemetamol images were also significantly higher than those of the wbPET ^18^F-FDG and ^18^F-flutemetamol images, except for the parietal SUVR of the ^18^F-FDG images (Table 2).

**TABLE 1.** Mean SUVRs of ^18^F-FDG and ^18^F-Flutemetamol Images Acquired Using dhPET and Conventional wbPET Scanners

Tracer	dhPET	wbPET
^18^F-FDG	1.17 ± 0.18	1.04 ± 0.14
^18^F-flutemetamol	2.09 ± 0.47	1.79 ± 0.46

**TABLE 2.** Regional SUVRs of ^18^F-FDG and ^18^F-Flutemetamol Images Acquired Using dhPET and Conventional wbPET Scanners

Tracer	Modality	Frontal	Temporal	Posterior cingulate	Parietal
^18^F-FDG	dhPET	1.25 ± 0.16*	1.10 ± 0.19*	1.23 ± 0.23*	1.00 ± 0.20
	wbPET	1.11 ± 0.14	1.02 ± 0.17	1.16 ± 0.22	1.04 ± 0.20
^18^F-flutemetamol	dhPET	2.03 ± 0.49*	1.86 ± 0.48*	2.08 ± 0.55*	1.78 ± 0.42*
	wbPET	1.69 ± 0.47	1.63 ± 0.45	1.83 ± 0.53	1.66 ± 0.43

* Significantly higher than wbPET (*P* < 0.05, Bonferroni adjustment).

**FIGURE 5.**
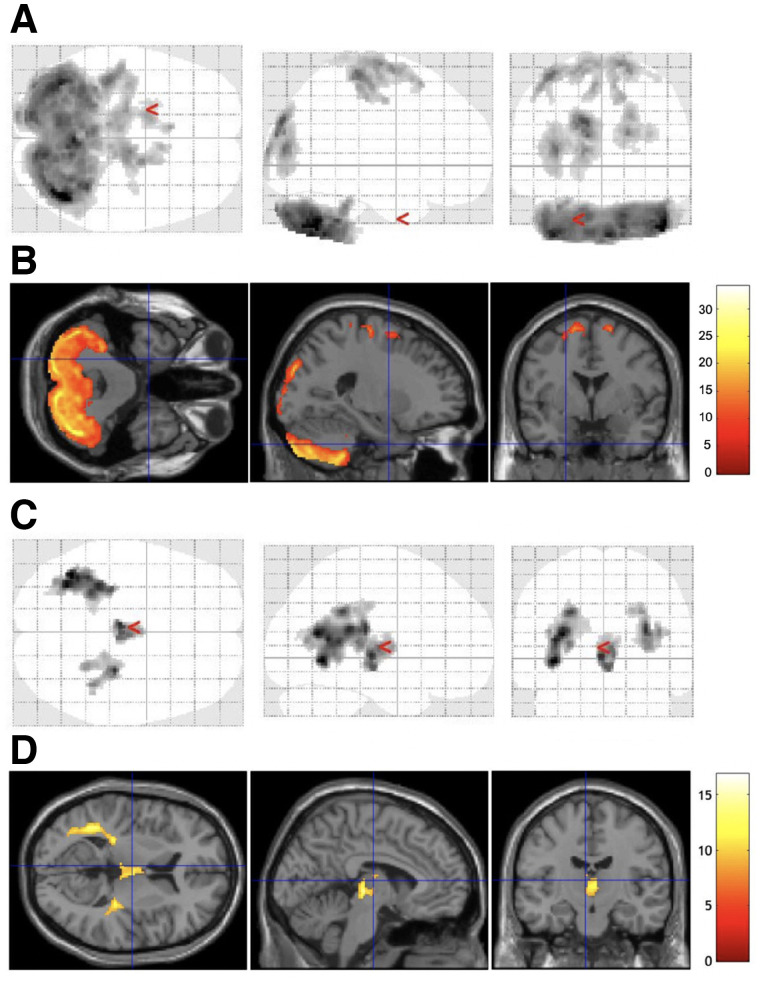
Areas of significant difference in ^18^F-FDG PET images between wbPET scanner and dhPET scanner. Glass brain (a display by SPM, which shows maximum-intensity projection [MIP] of the statistical map in 3 orthogonal planes) (A) and section (B) show higher accumulation in cerebellum, occipital lobe, and around central sulci in images acquired using wbPET scanner than in those acquired using dhPET scanner. Glass brain (C) and section (D) show higher peripheral ventricle accumulations in images acquired using dhPET scanner than in those acquired using wbPET scanner. Scale bar indicates *t* values.

**FIGURE 6.**
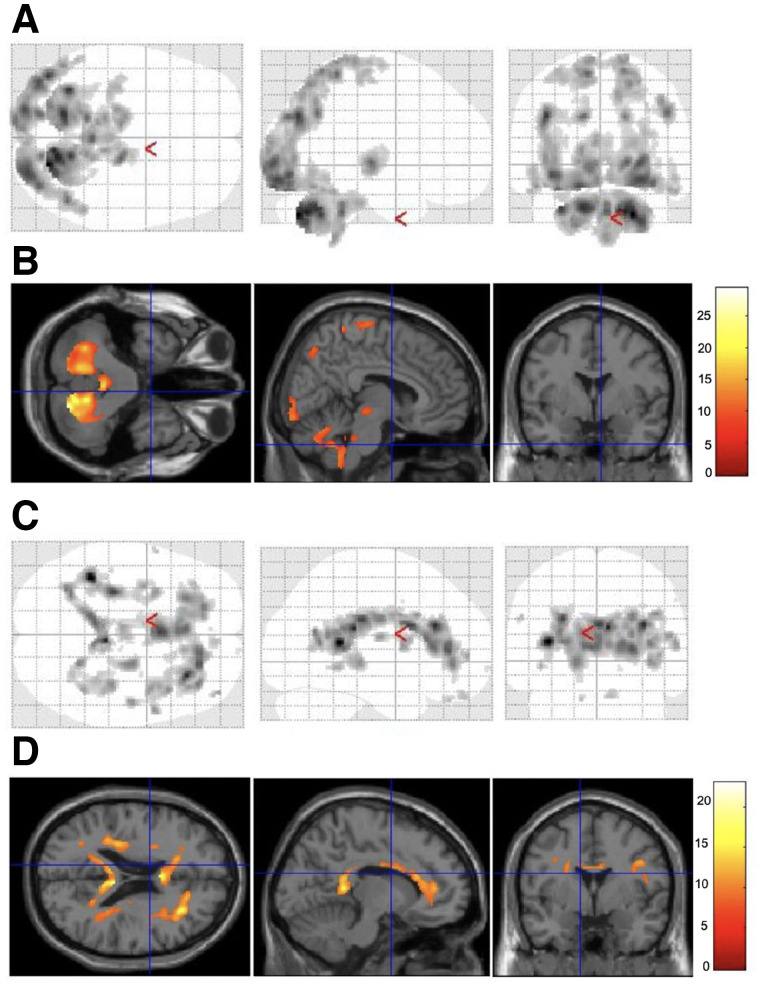
Areas of significant difference in ^18^F-flutemetamol PET images between wbPET scanner and dhPET scanner. Glass brain (a display by SPM, which shows maximum-intensity projection [MIP] of the statistical map in 3 orthogonal planes) (A) and section (B) show higher cerebellar and occipitoparietal accumulations in images acquired using wbPET scanner than in those acquired using dhPET scanner. Glass brain (C) and section (D) show higher peripheral ventricle area and frontal white matter accumulations in images acquired using dhPET scanner than in those acquired using wbPET scanner. Scale bar indicates *t* values.

Voxel-based analysis revealed that accumulations in the cerebellum, in the lateral occipital cortices, and around the central sulcus area were lower and that accumulations around the ventricle systems were higher in dhPET ^18^F-FDG images than in wbPET ^18^F-FDG images (Fig. 5). Accumulations in the cerebellar dentate nucleus, in the midbrain, in the lateral occipital cortices, and around the central sulcus area were higher in wbPET ^18^F-flutemetamol images than in dhPET ^18^F-flutemetamol images, whereas accumulations around the ventricle systems were higher in dhPET ^18^F-flutemetamol images than in wbPET ^18^F-flutemetamol images (Fig. 6).

## DISCUSSION

To our knowledge, this is the first clinical report on the application of a silicon photomultiplier TOF dhPET scanner. The high spatial resolution and low scatter noise of the scanner enable better detection of detailed cortical accumulations of ^18^F-FDG or ^18^F-flutemetamol. Clinically, this TOF-dhPET scanner with a silicon photomultiplier offers better resolution and contrast within the cortical distributions of PET tracers than do conventional PET scanners, perhaps because of the higher spatial resolution and lower scatter noise of dhPET than of wbPET, rather than higher statistical noise. One study highlighted that the ability to depict the red nuclei in brain ^18^F-FDG PET images is an indicator of high resolution (15). As shown in Figure 2, the red nucleus could clearly be visualized by the dhPET system, which may indicate its high resolution.

As shown in Figure 4, the high-resolution amyloid PET image accurately showed accumulations in the cortices, demonstrating the clinical impact of the method. Because of the spatial resolution and spillover of white matter uptake to the cortical ribbon limitations of wbPET, the increased uptake observed in the right temporal cortices appeared equivocal, which could lead to a misdiagnosis of positive accumulation. In contrast, dhPET imaging demonstrated the true accumulation (i.e., not increased accumulation). In the amyloid PET images, some subjects may show ambiguous accumulation, as observed in our subject, although this is not common. Therefore, a resolution equivalent to that of our PET system may be required to make an accurate diagnosis.

One advantage of the dhPET system is not needing to acquire a transmission scan for attenuation correction using an external radiation source, thus avoiding exposing the patient to external radiation. Even when additional imaging is required or multiple PET tracer images are repeatedly acquired of the same subject, frequent PET examinations are possible because of the lower radiation exposure using the dhPET system. However, as shown in Figures 5 and 6, we detected significant regional differences in accumulation distributions between wbPET and dhPET images. After familywise error correction, the occipital lobe and cerebellar accumulation counts of the dhPET images were lower than those of the wbPET images. Therefore, as long as this phenomenon is considered, the dhPET system may be used for routine clinical examination without the need for CT attenuation correction. However, to take advantage of the high resolution of the dhPET system and eliminate differences in accumulation distribution due to attenuation correction among different PET scanners, it will be necessary to create a database of healthy controls using this system.

When measuring the regional cortical SUVRs of glucose metabolism and amyloid deposition (16), high-resolution images obtained using the dhPET system will provide more accurate SUVRs and enable correct diagnoses. The mean cortical SUVRs calculated from the dhPET images were consistently higher than those calculated from the wbPET images. One reason may be that SUVR is obtained by dividing the cortical counts by the cerebellar counts, and the cerebellar counts of the dhPET images tended to be lower than those of the wbPET images (Figs. 5 and 6). The regional SUVRs of ^18^F-FDG and ^18^F-flutemetamol on the dhPET images were also significantly higher than those of ^18^F-FDG and ^18^F-flutemetamol on the wbPET images, except for the parietal SUVR of the ^18^F-FDG images. Patients with dementia have greater atrophy and hypometabolism in the parietal region than in other regions; therefore, we speculate that this difference impacted the counts of enlarged sulci, which are fewer on high-resolution dhPET images. Moreover, a large metabolism decrease would have further weakened the differences. The dhPET images had a lower partial-volume effect because of the high spatial resolution. The voxel-based analysis revealed lower accumulations in the ^18^F-FDG dhPET images than in the ^18^F-FDG wbPET images in the cerebellum, in the lateral occipital cortices, and around the central sulcus regions. This lower accumulation in the cerebellar and occipital regions is likely due to the attenuation correction method, whereas that around the central sulcus region is likely due to the high spatial resolution of the dhPET because the central sulci are wide in older adults. The accumulation differences near the ventricles may be related to partial-volume differences; however, this possibility could not be verified in our study. Additionally, differences in scanning time points may be a significant factor. Because dhPET scanning was always performed approximately 30 min after the wbPET scanning, this time difference may have affected the differences in ^18^F-FDG and ^18^F-flutemetamol accumulation and washout from the brain structures.

In an aging society, measures to combat dementia are crucial, and early diagnosis has the potential to delay or suppress the onset of dementia. Our findings will facilitate the widespread use of dhPET systems for scanning the brain to enable individuals to benefit from early diagnoses of dementia (17), epilepsy, and brain tumors.

There are several limitations to this study. Because the wbPET scanner was not a silicon photomultiplier PET system, one may argue that it would have high spatial resolution. However, the resolutions of the silicon photomultiplier PET/CT Discovery MI system, digital Biograph Vision PET/CT system, and Vereos PET/CT system are also approximately 4 mm in FWHM (18–21). Moreover, even if the resolution of the silicon photomultiplier PET scanner is compared with that of the wbPET scanner, there will not be a significant impact on the results of this study because the resolution of the dhPET system is less than 3 mm in FWHM (10). Furthermore, even if the silicon photomultiplier is compared with previous or current head-only–designed PET scanners (e.g., HRRT and helmet-type PET scanners) or with approaches that correct for partial-volume errors or improve segmentation using PET/MRI (22), the results are unlikely to change significantly.

The dhPET scan was always acquired after the standard system scan. This sequence may introduce bias related to the timing window of the acquired PET images after the injection. We suspect that the SUVR differences between the systems are not due simply to differences in scan timing but to a combination of differences in scan time and attenuation correction methods. Moreover, it is worth highlighting that an increase in SUVR using the dhPET does not represent clinical or technical superiority over wbPET.

Statistical image analysis with a database of healthy controls using this system would help with clinical diagnoses in routine practice. However, interpreting data acquired using this system would remain challenging if there were major differences in attenuation correction relative to PET/CT.

To take advantage of the high spatial resolution of the dhPET system, it is necessary to reduce the effects of head motion during the 20–30 min of scanning. To address this issue, it will be crucial to optimize the conditions for generating high-quality images in a short period and to develop image reconstruction methods that detect or account for head motion.

## CONCLUSION

Our novel dhPET scanner can provide high-resolution and high-sensitivity images for ^18^F-FDG and amyloid PET that are superior to those offered by conventional wbPET. This new technology will enable more accurate diagnoses of brain diseases in the future.

## DISCLOSURE

Kazunari Ishii received a research grant from Shimadzu Corp.; Kazunari Ishii reports that the dhPET scanner was provided by Shimadzu Corp.; and Yoshiyuki Yamakawa, Suzuka Minagawa, Shiho Takenouchi, Atsushi Ohtani, and Tetsuro Mizuta are employees of Shimadzu Corp. No other potential conflict of interest relevant to this article was reported.

## Notes

KEY POINTS **QUESTION:** Do ^18^F-FDG and amyloid PET images obtained with a TOF dhPET system have clinical superiority to those obtained with a conventional wbPET system? **PERTINENT FINDINGS:** ^18^F-FDG and amyloid images acquired using dhPET are superior in spatial resolution to those acquired using wbPET and provide an accurate diagnosis. The mean SUVRs of ^18^F-FDG and amyloid dhPET images are significantly higher than those of ^18^F-FDG and amyloid wbPET images, respectively. **IMPLICATIONS FOR PATIENT CARE:** ^18^F-FDG and amyloid images acquired using dhPET provide useful information and support accurate diagnosis for individual patients.
